# Editorial: Women in plant biotechnology 2022

**DOI:** 10.3389/fpls.2023.1292441

**Published:** 2023-10-13

**Authors:** Rose A. Marks, Jill M. Farrant

**Affiliations:** ^1^ Department of Horticulture, Michigan State University, East Lansing, MI, United States; ^2^ Plant Resilience Institute, Michigan State University, East Lansing, MI, United States; ^3^ Department of Molecular and Cell Biology, University of Cape Town, Rondebosch, South Africa

**Keywords:** diversity, equity, women, plant science, biotechnology

Maximizing diversity promotes the growth and maintenance of vibrant, productive, and resilient systems Montgomery, 2020. This is true of both the biological systems we study and the human systems we work within. Unfortunately, diversity is rarely maximized within the socioeconomic landscape of scientific research, and many perspectives and identities remain severely underrepresented in science. Noteworthy biases and inequities exist in research communities along national, racial, class, and gender axes (West et al., 2013; Holman et al., 2018; Amarante et al., 2021; Hotaling et al., 2021; Maas et al., 2021; Madzima and MacIntosh, 2021; Marks et al., 2021, 2023). At present, fewer than 30% of researchers worldwide are women, and plant biology is no exception to this trend (Marks et al., 2023). Long-standing biases and gender stereotypes have excluded women from science-related fields, collaboration networks, and prestigious leadership positions (West et al., 2013; Frances et al., 2020; Madzima and MacIntosh, 2021; Lerman et al., 2022), and progress toward a gender-balanced research environment has been painfully slow. Despite substantial and meaningful scientific contributions by women throughout history, gender biases persist in hiring, funding decisions, and citation rates (Larivière et al., 2013; Fox et al., 2016; Bonham and Stefan, 2017; Holman et al., 2018; Witteman et al., 2019; Frances et al., 2020; Wapman et al., 2022). These factors, in addition to more subtle biases, contribute to the widespread underrepresentation of women in science.

In order to create a more equitable and inclusive discipline, gender equality must be promoted, stereotypes defeated, and girls and women should be encouraged to participate in research and other scientific activities. This Research Topic, “*Women in Biotechnology*”, aims to do just that–to promote the work of women scientists across the globe in plant biotechnology. Despite the importance of this topic and the undeniable talent of women scientists, very few articles have been submitted to this Research Topic. Furthermore, some of the submissions received were led by male authors and therefore excluded. As a result, the final collection contains only four articles. While it is difficult to determine the reason behind the low submission number, we speculate that it is a symptom of the overall underrepresentation of women in science. Alternatively, it is possible that some women authors preferred to publish in subject-specific collections, journals, or other topics that are not directly related to their gender identity. Still, the four articles included in this Research Topic highlight the talent, diversity, and ingenuity of women scientists.

The articles in this Research Topic fall into two distinct categories. The first two articles describe transgenic manipulations to overexpress and silence genes of interest, while the second two articles describe methodological advances that increase the efficiency of transformation protocols, a major bottleneck in plant biotechnology.

## Transgene expression

The papers by Kopertekh and Reichardt and Kiełbowicz‐Matuk et al. describe important phenotypic consequences of transgenic manipulations on agronomically relevant traits, including biomass, flowering time, and stress tolerance. Kopertekh and Reichardt show that the transient expression of a cell cycle regulatory gene (At-CDC27a) leads to enlarged cells, increased protein accumulation, and overall elevated biomass in *Nicotiana benthamiana*. Kiełbowicz‐Matuk et al. have overexpressed and also silenced a clock-controlled gene encoding a B-box protein (StBBX24) in *Solanum tuberosum*. They show that silenced lines flowered earlier than wild-type plants, while overexpressing plants did not flower at all. Overexpressing lines also exhibited substantial modifications to the expression of downstream genes in flowering pathways, while silenced lines showed a reduction in salt tolerance, lower antioxidant activity, and decreased Na+ transporter expression. Both of these studies are exciting success stories of single-gene work with important downstream consequences for traits with agronomic value. Work in this area has promising applications for improving food security, sustainability, and agronomic resilience.

## Methodological advances

The second set of papers by Wang et al. and Monroy-Borrego and Steinmetz provide useful methodological advances that can be used to accelerate plant transformation. Wang et al. describe a new protocol to transform wheat via particle bombardment. They present a carefully optimized methodology along with detailed instructions for performing particle bombardment on wheat. They also include customized recommendations and troubleshooting advice. Monroy-Borrego and Steinmetz describe three methods (mechanical, foliar spray, and petiole and stem injection) for initiating tobacco mosaic virus infection in *Nicotiana benthamiana*. Each of these three methods offers different advantages – mechanical application is highly reproducible, foliar spray is scalable in agricultural settings, and syringe inoculation is aseptic and may therefore be suitable for the pharmaceutical industry. Plant transformation is a major bottleneck in plant biotechnology, and the improved approaches presented in these two studies could help advance the field and overcome current roadblocks.

## Conclusion

Women are doing excellent work across biotechnology, from validating important genes to developing improved tools and techniques. However, they may not be gaining the recognition or promotion their men counterparts enjoy. As indicated above, in the plant sciences alone, a considerable majority of articles are authored by men, who receive more citations and generally have access to increased funding opportunities compared to their women counterparts. Considerable and active efforts to engage with and highlight women in science are important steps toward increasing equity in the field of plant biotechnology.

## Author contributions

RM: Conceptualization, Writing – original draft. JF: Conceptualization, Writing – review & editing.

## References

[B1] Amarante V. Burger R. Chelwa G. Cockburn J. Kassouf A. McKay A.. (2021). Underrepresentation of developing country researchers in development research. Appl. economics Lett. 29, 1–6. doi: 10.1080/13504851.2021.1965528

[B2] Bonham K. S. Stefan M. I. (2017). Women are underrepresented in computational biology: An analysis of the scholarly literature in biology, computer science and computational biology. PloS Comput. Biol. 13, e1005134. doi: 10.1371/journal.pcbi.1005134 29023441 PMC5638210

[B3] Fox C. W. Burns C. S. Muncy A. D. Meyer J. A. (2016). Gender differences in patterns of authorship do not affect peer review outcomes at an ecology journal. Funct. Ecol. 30, 126–139. doi: 10.1111/1365-2435.12587

[B4] Frances D. N. Fitzpatrick C. R. Koprivnikar J. McCauley S. J. (2020). Effects of inferred gender on patterns of co-authorship in ecology and evolutionary biology publications. Bull. Ecol. Soc. America 101. doi: 10.1002/bes2.1705

[B5] Holman L. Stuart-Fox D. Hauser C. E. (2018). The gender gap in science: How long until women are equally represented? PloS Biol. 16, e2004956. doi: 10.1371/journal.pbio.2004956 29672508 PMC5908072

[B6] Hotaling S. Kelley J. L. Frandsen P. B. (2021). Toward a genome sequence for every animal: Where are we now? Proc. Natl. Acad. Sci. United States America 118 (52) e2109019118. doi: 10.1073/pnas.2109019118 PMC8719868 34862323

[B7] Larivière V. Ni C. Gingras Y. Cronin B. Sugimoto C. R. (2013). Bibliometrics: global gender disparities in science. Nature 504, 211–213. doi: 10.1038/504211a 24350369

[B8] Lerman K. Yu Y. Morstatter F. Pujara J. (2022). Gendered citation patterns among the scientific elite. Proc. Natl. Acad. Sci. United States America 119, e2206070119. doi: 10.1073/pnas.2206070119 PMC9546584 36161888

[B9] Maas B. Pakeman R. J. Godet L. Smith L. Devictor V. Primack R. (2021). Women and Global South strikingly underrepresented among top-publishing ecologists. Conserv. Lett. 14. doi: 10.1111/conl.12797

[B10] Madzima T. F. MacIntosh G. C. (2021). Equity, diversity, and inclusion efforts in professional societies: intention versus reaction. Plant Cell 33, 3189–3193. doi: 10.1093/plcell/koab186 34264320 PMC8505871

[B11] Marks R. A. Amézquita E. J. Percival S. Rougon-Cardoso A. Chibici-Revneanu C. Tebele S. M.. (2023). A critical analysis of plant science literature reveals ongoing inequities. Proc. Natl. Acad. Sci. United States America 120, e2217564120. doi: 10.1073/pnas.2217564120 PMC10013813 36853942

[B12] Marks R. A. Hotaling S. Frandsen P. B. VanBuren R. (2021). Representation and participation across 20 years of plant genome sequencing. Nat. plants 7, 1571–1578. doi: 10.1038/s41477-021-01031-8 PMC8677620 34845350

[B13] Montgomery B. L. (2020). Planting equity: using what we know to cultivate growth as a plant biology community. Plant Cell 32, 3372–3375. doi: 10.1105/tpc.20.00589 32873632 PMC7610297

[B14] Wapman K. H. Zhang S. Clauset A. Larremore D. B. (2022). Quantifying hierarchy and dynamics in US faculty hiring and retention. Nat. 610, 120–127. doi: 10.1038/s41586-022-05222-x PMC9534765 36131023

[B15] West J. D. Jacquet J. King M. M. Correll S. J. Bergstrom C. T. (2013). The role of gender in scholarly authorship. PloS One 8, e66212. doi: 10.1371/journal.pone.0066212 23894278 PMC3718784

[B16] Witteman H. O. Hendricks M. Straus S. Tannenbaum C. (2019). Are gender gaps due to evaluations of the applicant or the science? A natural experiment at a national funding agency. Lancet 393, 531–540. doi: 10.1016/S0140-6736(18)32611-4 30739688

